# Membrane-Bound Protease FtsH Protects PhoP from the Proteolysis by Cytoplasmic ClpAP Protease in *Salmonella* Typhimurium

**DOI:** 10.4014/jmb.2306.06016

**Published:** 2023-06-17

**Authors:** Hyungkeun Song, Eunna Choi, Eun-Jin Lee

**Affiliations:** Department of Life Sciences, School of Life Sciences and Biotechnology, Korea University, Seoul 02841, Republic of Korea

**Keywords:** FtsH protease, ClpAP protease, PhoP/PhoQ two-component system

## Abstract

Among the AAA+ proteases in bacteria, FtsH is a membrane-bound ATP-dependent metalloprotease, which is known to degrade many membrane proteins as well as some cytoplasmic proteins. In the intracellular pathogen *Salmonella enterica* serovar Typhimurium, FtsH is responsible for the proteolysis of several proteins including MgtC virulence factor and MgtA/MgtB Mg^2+^ transporters, the transcription of which is controlled by the PhoP/PhoQ two-component regulatory system. Given that PhoP response regulator itself is a cytoplasmic protein and also degraded by the cytoplasmic ClpAP protease, it seems unlikely that FtsH affects PhoP protein levels. Here we report an unexpected role of the FtsH protease protecting PhoP proteolysis from cytoplasmic ClpAP protease. In FtsH-depleted condition, PhoP protein levels decrease by ClpAP proteolysis, lowering protein levels of PhoP-controlled genes. This suggests that FtsH is required for normal activation of PhoP transcription factor. FtsH does not degrade PhoP protein but directly binds to PhoP, thus sequestering PhoP from ClpAP-mediated proteolysis. FtsH's protective effect on PhoP can be overcome by providing excess ClpP. Because PhoP is required for *Salmonella*'s survival inside macrophages and mouse virulence, these data implicate that FtsH's sequestration of PhoP from ClpAP-mediated proteolysis is a mechanism ensuring the amount of PhoP protein during *Salmonella* infection.

## Introduction

**Fig. 1:**
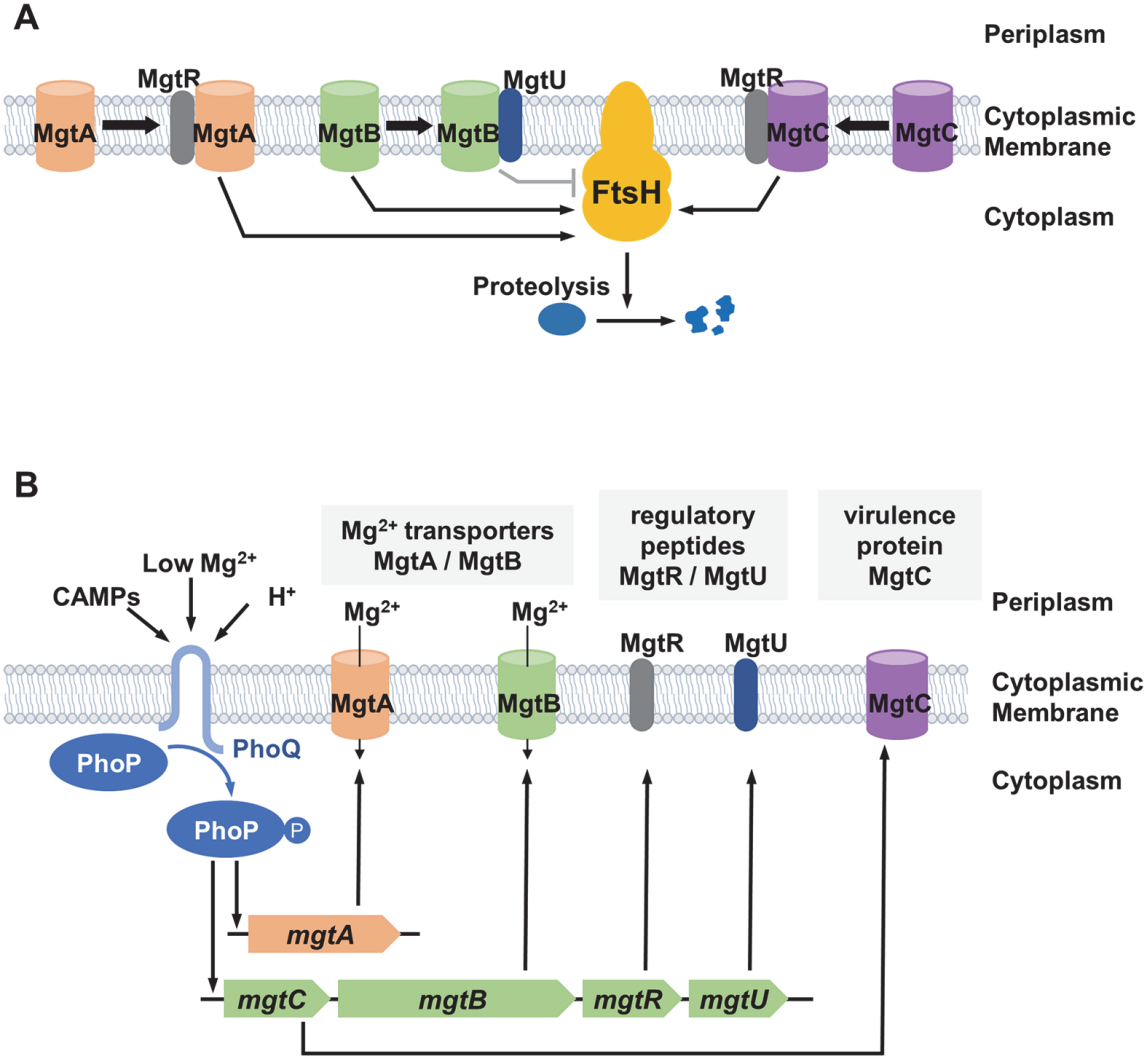
Membrane-bound FtsH controls proteolysis of PhoP-regulated genes in *Salmonella* Typhimurium. (**A**) *Salmonella* AAA+ protease FtsH is involved in proteolysis of various proteins including MgtA Mg^2+^ transporter, MgtB Mg^2+^ transporter, and MgtC virulence protein. Small protein MgtR interacts with MgtC and MgtA proteins to guide FtsH to degrade MgtC and MgtA proteins. Other small protein MgtU binds to MgtB and protects it from the degradation by FtsH. (**B**) The PhoP/PhoQ two-component regulatory system detects the low concentration of Mg^2+^, acidic pH, and antimicrobial peptides inside *Salmonella*-containing vesicle and activates transcription of the *phoP*-activated genes encoding the above-mentioned membrane proteins.

AAA+ proteases are ATP-dependent proteases (AAA+; ATPases associated with diverse cellular activities) that play important roles in cellular survivals. They degrade damaged or misfolded proteins to maintain the protein homeostasis. Proteolytic process consists of ATP-dependent unfolding of target proteins by the ATPase subunit and translocation of target protein into the proteolytic chamber of protease domain [1]. In bacteria, there are five different AAA+ proteases, including ClpAP, ClpXP, HslUV, Lon, and FtsH. Unlike other proteases located in the cytoplasm, FtsH protease is the membrane-bound protease that is anchored via two transmembrane helices. Because FtsH degrades many membrane proteins and some of cytoplasmic proteins as substrates [2, 3], it is involved in many biological processes including regulation of heat shock response, biosynthesis of lipopolysaccharide, and stress response [2]. It was reported that the *ftsH* gene is essential in several bacteria [2], possibly due to its role in maintaining membrane protein homeostasis. In intracellular pathogen *Salmonella enterica* serovar Typhimurium, FtsH controls proteolysis of additional proteins, whose functions are required during infection. These include MgtC virulence factor responsible for pH or Pi homeostasis and MgtB/MgtA Mg^2+^ transporters for acquisition of Mg^2+^ ion [4, 5] (Fig. 1A), suggesting that FtsH-mediated protein quality control appears to be associated with *Salmonella* pathogenesis. Small membrane peptides often guide this FtsH-mediated proteolysis. For example, MgtR promotes FtsH-mediated proteolysis of both MgtC and MgtA Mg^2+^ transporter and MgtU protects MgtB Mg^2+^ transporter from the FtsH-mediated proteolysis [4-6].

Interestingly, transcription of genes (*mgtC*, *mgtB*, and *mgtA*) encoding all aforementioned FtsH substrates are controlled by the PhoP/PhoQ two-component system. The two-component system is a regulatory signal-transduction system in bacteria, which contains two conserved components of protein kinase and response regulatory protein. Mostly it involves phosphotransfer reactions inducing conformational or chemical changes in the regulatory domain that leads to the activation of affected proteins [7]. S. Typhimurium operates the PhoP/PhoQ two-component regulatory system that governs virulence and the adaptation to environment in which the magnesium ion is limited [8]. It is also essential for *Salmonella* to survive and replicate inside the host cell [9]. The PhoP/PhoQ system consists of inner membrane sensor protein PhoQ and the cytoplasmic regulator PhoP. Low Mg^2+^ as well as acidic pH and antimicrobial peptides outside the cell promote autophosphorylation of PhoQ and phosphorylation of PhoP by phospho-PhoQ [10, 11]. Phosphorylated PhoP then induces transcription of several genes encoding Mg^2+^ transporters and virulence proteins, including the *mgtA* gene and *mgtCBRU* operon (Fig. 1B) [8]. MgtA and MgtB are Mg^2+^ transporters that play an important role in maintaining Mg^2+^ homeostasis [12]. MgtC is the virulence protein required for growth in low Mg^2+^ environment and intramacrophage survival [13]. MgtR is the small regulatory protein that assists degradation of MgtC and MgtA Mg^2+^ transporter by FtsH [5, 6]. Other small protein MgtU binds to MgtB Mg^2+^ transporter and prevents MgtB from FtsH-mediated degradation [4].

Despite the fact that FtsH breaks down a number of membrane proteins encoded by PhoP-regulated genes, little is known about the relationships between PhoP and the membrane-bound FtsH protease. PhoP itself is known to be degraded by the cytoplasmic ClpAP protease [14]. In this paper, we report a novel mechanism ensuring the transcription activity of PhoP by the FtsH protease. FtsH is required for normal expression of PhoP-regulated gene products including PhoP. However, FtsH does not degrade PhoP, rather FtsH protects PhoP from ClpAP protease by directly binding to PhoP protein. This sequestration by the membrane-bound FtsH protease provide a “safeguard” mechanism to maintain amounts of PhoP that might be required during *Salmonella* infection.

## Materials and Methods

### Bacterial Strains, Oligonucleotides, and Growth Conditions

Bacterial strains used in this study are listed in Table S1. All *Salmonella enterica* serovar Typhimurium strains are derived from the wild-type strain 14028s [23] and were constructed using l-mediated recombination [24] and phage P22-mediated transduction [25] as described. All DNA oligonucleotides are listed in Table S2. Bacteria were grown at 37°C in Luria-Bertani broth (LB), N-minimal medium (pH 7.7) [26] supplemented with 0.1%casamino acids, 38 mM glycerol and the indicated concentrations MgCl_2_. Ampicillin was used at 50 μg/ml, kanamycin at 50 μg/ml, chloramphenicol at 25 μg/ml, and tetracycline at 10 μg/ml. Isopropyl-β-D thiogalactopyranoside (IPTG) was used at 0.0626 mM or 0.1 mM.

### Plasmid Construction

The plasmids pBAD33-*clpP*-6×*His*, pBAD33-*ftsH*-*FLAG*, pUHE21-*ftsH*_*A* (AAA+ domain), pUHE21-*ftsH*_*P* (protease domain), and pUHE21-*ftsH*_*AP* (cytoplasmic domain) were constructed as follows: DNA fragments corresponding to the *clpP*, *ftsH*, *ftsH*_*A*, *ftsH*_*P*, and *ftsH*_*AP* genes were generated by PCR with the primer pairs PHK139 / PHK140 (for pBAD33-*clpP*-6×*His*), KHU901 / KHU902 (for pBAD33-*ftsH*-*FLAG*), PHK169 / PHK170 (for pUHE21-*ftsH*_*A*), PHK171 / PHK172 (for pUHE21-*ftsH*_*P*), PHK169 / PHK172 (for pUHE21-*ftsH*_*AP*)and 14028s genomic DNA as a template. The amplified DNA fragments were digested with KpnI / XbaI (for pBAD33-*clpP*-6×*His*), XbaI / HindIII (for pBAD33-*ftsH*-*FLAG*), and BamHI / HindIII (for pUHE21-*ftsH*_*A*, pUHE21-*ftsH*_*P*, and pUHE21-*ftsH*_*AP*) and cloned into pBAD33 and pUHE21 plasmids digested with the same enzymes, respectively.

The plasmids pBAD33-*ftsH*^H414A^-*FLAG*, pBAD33-*ftsH*^E415A^-*FLAG*, pBAD33-*ftsH*^H418A^-*FLAG* were constructed as follows: DNA fragments carrying various nucleotide substitutions in the *ftsH* gene were generated by a two-step PCR process. For the first PCR reaction, we used two primer pairs KHU901 / KHU494 and KHU893 / KHU902 (for 414^th^ histidine codon to alanine), KHU901 / KHU896 and KHU895 / KHU902 (for 415^th^ glutamic acid codon to alanine), KHU901 / KHU898 and KHU897 / KHU902 (for 418^th^ histidine codon to alanine), and 14028s wild-type genomic DNA as a template. The amplified DNA fragments were digested with XbaI / HindIII and cloned into pBAD33 plasmids digested with the same enzymes.

### Construction of Strains with the Chromosomal Deletions or C-Terminally Tagged Genes

*Salmonella* strains deleted for the *clpX* or *clpS* genes and strains with HA-tagged *mgtR* and 8×*Myc*-tagged *mgtA* genes were generated by the one-step gene inactivation method [24]. Km^R^ cassettes for the *clpX* gene, *clpS* gene, *mgtR*-*HA*, and *mgtA*-8×*Myc* genes were PCR amplified from plasmid pKD4 using primers PHK053 / PHK054 (*clpX* deletion), PHK167 / PHK168 (*clpS* deletion), PHK072 / PHK073 (*mgtR*-*HA* tagging), PHK114 / PHK115 (*mgtA*-8×*Myc* tagging) and the resulting PCR products were integrated into the 14028s chromosome to generate HK118 (Δ*clpX*::Km^R^), *mgtR*-*HA*-Km^R^, and HK161 (*mgtA*-8×*Myc*-Km^R^) or the EN1573 chromosome (*phoP*-*HA*) to generate HK251 (*phoP*-*HA* Δ*clpS*::Km^R^). Δ*clpX* (HK122), *mgtR*-*HA* (HK135), and *mgtA*-8×*Myc* (HK162) strains were generated by removing Km^R^ cassettes from each strain via plasmid pCP20 as described [24].

Because the *ftsH* gene encoding the FtsH protease is essential in S. Typhimurium, pUHE21-*ftsH* plasmid was introduced into strains HK084 (Δ*clpA*), HK087 (Δ*hslUV*), HK135 (*mgtR*-*HA*), HK162 (*mgtA*-8×*Myc*), and EN1573 (*phoP*-*HA*) and then P22 phage lysates grown in EN656 (Δ*ftsH*::Km^R^ / pUHE21-*ftsH*) were used to transduce each strain. Strains HK094 (Δ*clpA*, Δ*ftsH*::Km^R^ / pUHE21-*ftsH*), HK097 (Δ*hslUV*, Δ*ftsH*::Km^R^ /pUHE21-*ftsH*), HK140 (*mgtR*-*HA*, Δ*ftsH*::Km^R^ / pUHE21-*ftsH*), HK164 (*mgtA*-8×*Myc*, Δ*ftsH*::Km^R^ / pUHE21-*ftsH*), and HK202 (*phoP*-*HA*, Δ*ftsH*::Km^R^ / pUHE21-*ftsH*) were generated via selection for kanamycin resistance.

### Western Blot Analysis

Bacteria were grown in 10 ml N-minimal medium containing 10 mM or 0.01 mM Mg^2+^ to OD_600_= 0.5. Crude extracts were prepared in TBS (Tris-buffered saline) buffer by sonication, electrophoresed on 12% SDS-polyacrylamide gel and transferred to PVDF membranes. The MgtB, MgtC, and Fur proteins were detected using anti-MgtB, anti-MgtC, and anti-Fur polyclonal antibodies as described [27]. HA, FLAG, 6×*His*, and 8×*Myc* tagged proteins were detected using anti-HA, anti-*FLAG*, anti-His polyclonal antibodies, and anti-Myc monoclonal antibody respectively. The blots were incubated with above antibodies for overnight as primary antibodies, followed by incubation with anti-rabbit or anti-mouse IgG horseradish peroxidase-linked antibodies (1:10,000 dilution, ThermoFisher, USA) for 1 h. And then the blots were detected using SuperSignal West Femto Maximum Sensitivity Substrate (ThermoFisher) or EZ-Western Lumi Pico (DoGenBio, South Korea). The data are representative of at least two independent experiments, which gave similar results.

### RNA Extraction and Quantitative Real-Time Polymerase Chain Reaction (RT-PCR)

Total RNA was isolated using RNeasy Kit (Qiagen, Germany) according to the manufacturer's instructions. The purified RNA was quantified using a Nanodrop machine (Thermo Scientific). cDNA was synthesized using PrimeScript RT reagent Kit (TaKaRa, Japan). The mRNA levels of *mgtB*, *phoP*, and *rrsH* genes were measured by quantification of cDNA using SYBR green PCR Master Mix (Applied Biosystems, USA) with appropriate primers and monitored using a StepOnePlus Real-Time PCR system (Applied Biosystems). Data were normalized to the levels of 16S ribosomal RNA amplified with appropriate primers.

### Immunoprecipitation Assay

The interaction between the PhoP and FtsH proteins was investigated in *Salmonella* strains expressing the *ftsH* gene from an arabinose-inducible plasmid (pBAD33 as a negative control or pBAD33-*ftsH*-*FLAG*) and the HA-tagged *phoP* gene from its chromosome (wild-type strain as a negative control or *phoP*-*HA* strain). Cells were grown overnight in N-minimal media containing 10 mM Mg^2+^. A 1/100 dilution of the bacterial culture was inoculated in 30 ml of N-minimal media containing 0.01 mM Mg^2+^ and grown for 4 h. Cells were then induced with 0.5 mM L-arabinose and grown for 1 h. Cells were normalized by measuring OD_600_. Crude extracts were prepared in TBS (Tris-buffered Saline) buffer by sonication. For a pull-down assay with anti-HA and anti-*FLAG* antibodies, 100 μl of the crude extracts were kept for input and 350 μl each of the crude extracts were mixed with 25 μl of EZview Red Anti-HA Affinity Gel (Sigma-Aldrich, USA) or EZview Red Anti-*FLAG* M2 Affinity Gel (Sigma-Aldrich) respectively for overnight at 4°C on nutator (BenchMark). After washing the beads, the bound proteins were eluted in SDS sample buffer, separated on a 12% SDS-polyacrylamide gel, and analyzed by Western blotting using anti-HA (1:5,000 dilution, Sigma-Aldrich) and anti-*FLAG* (1:5,000 dilution, Sigma-Aldrich) antibodies for overnight. The blots were washed and hybridized with anti-rabbit IgG horseradish peroxidase-linked antibodies (1:10,000 dilution, ThermoFisher) for 1 h and detected using SuperSignal West Femto Maximum Sensitivity Substrate (ThermoFisher) or EZ-Western Lumi Pico (DoGenBio).

## Results

### MgtB Protein Levels Dramatically Decrease When FtsH Is Depleted

**Fig. 2:**
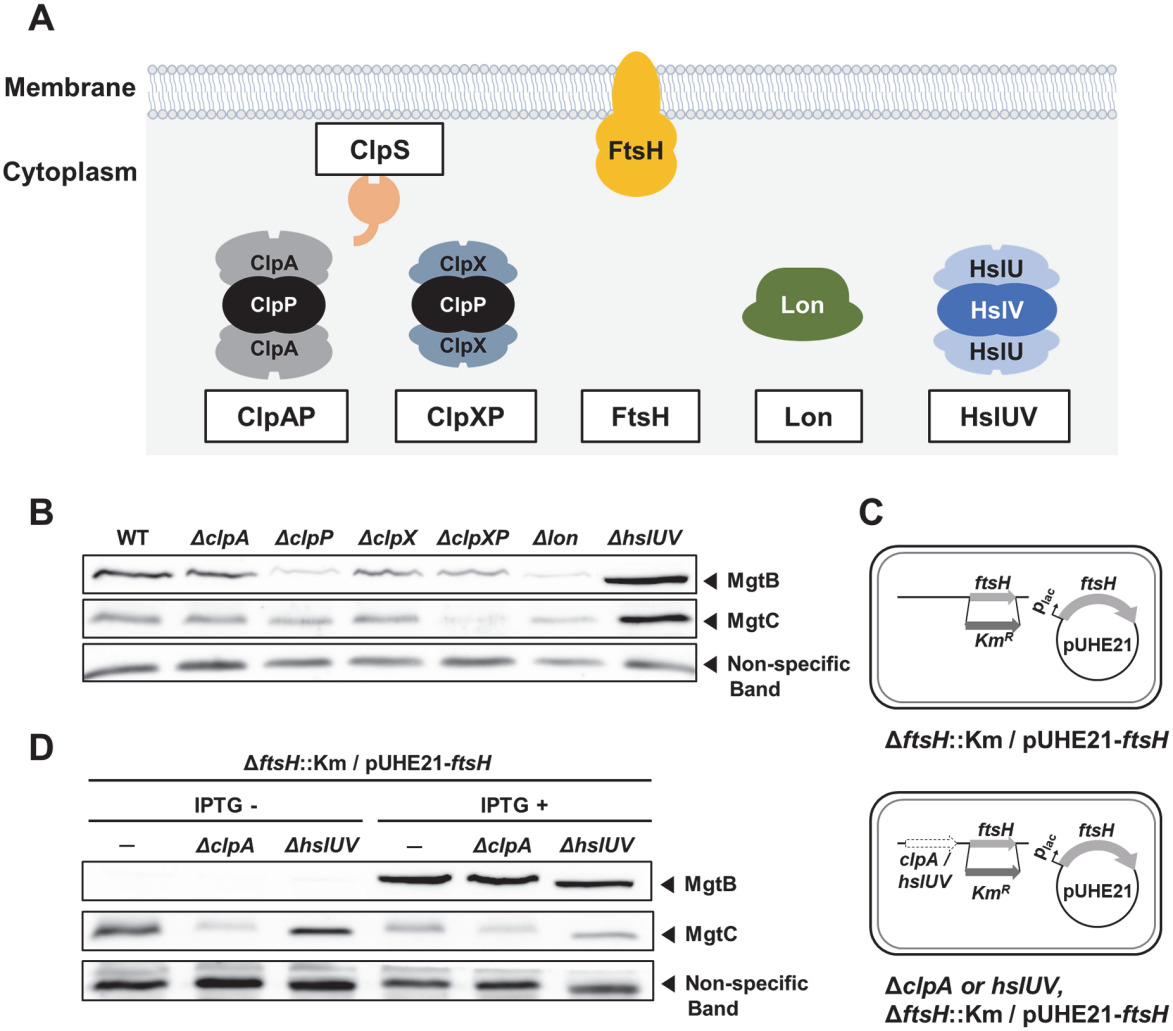
MgtB protein levels decrease when FtsH protease is depleted. (**A**) Schematic diagram of *Salmonella* AAA+ proteases. ClpAP, ClpXP, Lon, and HslUV are cytoplasmic proteases while FtsH is the only membrane-bound protease. ClpS is an adaptor protein for ClpAP protease. (**B**) Western blot analysis of crude extracts prepared from wild-type (14028s), Δ*clpA* (HK084), Δ*clpP* (HK081), Δ*clpX* (HK122), Δ*clpXP* (HK082), Δlon (HK083), and Δ*hslUV* (HK087) strains. Cells were grown to OD_600_ = 0.5 at 37°C in N-minimal medium containing 0.01 mM Mg^2+^. The samples were analyzed by anti-MgtB and anti-MgtC antibodies. Non-specific bands detected by MgtC polyclonal antibodies were used as loading controls. (**C**) Schematic cartoon of the *ftsH* conditional knockout mutant with the wild-type (EN656), Δ*clpA* (HK094), or Δ*hslUV* (HK097). (**D**) Western blot analysis of crude extracts prepared from the *ftsH* conditional knockout mutants with the wild-type (EN656), Δ*clpA* (HK094), or Δ*hslUV* (HK097) strains in the absence or presence of IPTG (0.0625 mM). Cells were grown to OD_600_ = 0.5 at 37°C in Nminimal medium containing 0.01 mM Mg^2+^. The samples were analyzed by anti-MgtB and anti-MgtC antibodies. Non-specific bands detected by MgtC polyclonal antibodies were used as loading controls.

In *Salmonella*, it contains five AAA+ proteases: ClpAP, ClpXP, Lon, HslUV, and FtsH (Fig. 2A) [1]. The ClpP protease couples with two different ATP-binding subunits, ClpA or ClpX, which unfold substrate proteins and translocate them to the ClpP protease [1]. In *Salmonella*, PhoP virulence regulator is known to be one of substrates of ClpAP protease. ClpS adaptor protein binds to substrates such as PhoP and assists the degradation by ClpAP [14]. Lon protease mediates selective degradation of misfolded proteins as well as other proteins [15]. HslUV is a protease complex that consists of ATPase subunit HslU and protease subunit HslV [15]. Among them, FtsH is the only membrane-bound AAA+ protease in *Salmonella*, which degrades both cytoplasmic and membrane proteins [2].

To assess amounts of MgtB and MgtC proteins in the *Salmonella* strains lacking respective proteases, we created strains deleted for *clpA*, *clpP*, *clpX*, *clpXP*, *lon*, *hslUV*, or *clpS* genes encoding cytoplasmic proteases or its regulatory subunits (Figs. 2A and S1). Because FtsH is an essential gene for bacteria to survive and grow, we used the *ftsH* conditional knockout strain that is deleted for the chromosomal *ftsH* gene and contains a plasmid with the *ftsH* gene under the IPTG-inducible promoter [16]. When we measured MgtB protein levels, strains lacking either ClpP or Lon proteases or a strain depleting FtsH decreased MgtB protein levels, while *hslUV* mutant strain increased MgtB levels (Figs. 2B and 2D). The decrease in MgtB levels in the *lon* mutant makes sense because Lon degrades H-NS responsible for silencing PhoP-mediated transcriptional activation [17]. And thus the *lon* mutant expected to maintain high levels of H-NS, strengthening repression of the PhoP-controlled genes. However, it was unlikely to detect the decreases in MgtB levels in the *clpP* or *ftsH* depletion. Because ClpP protease degrades PhoP responsible for *mgtB* transcription [14] and thus the *clpP* mutant was expected to show elevated MgtB levels. Similarly, FtsH is involved in degradation of most membrane proteins [2] and MgtA Mg^2+^ transporter, an MgtB homolog, seemed to be also degraded by FtsH protease [6]. Thus, MgtB Mg^2+^ transporter was expected to be a substrate for FtsH-mediated proteolysis. Additionally, a previous study using a temperature-sensitive *E. coli* *ftsH* mutant strain heterologously expressing HA-tagged *Salmonella* MgtB protein suggested that MgtB-HA protein might be degraded by the FtsH protease [4]. However, this was not the case in our experiments. MgtB proteins were accumulated when FtsH was replete but disappeared when FtsH was depleted (Fig. 2D). Further genetic combinations of *clpA* or *hslUV* mutations with the *ftsH* conditional knockout did not change MgtB’s behaviors in the FtsH-depleting condition (Figs. 2C and 2D).

Among these patterns, we focused MgtB’s behavior in the FtsH-depleted strain because it differs from MgtC’ behavior (Fig. 2D) even though the MgtC and MgtB proteins are a part of the same *mgtCBRU* operon (Fig. 1B) and its transcription is controlled by the same transcription factor PhoP. For MgtC’s behavior, one can consider additional factor, MgtR regulatory peptide, because MgtR is also produced from the same *mgtCBRU* operon, but MgtR promotes FtsH-mediated MgtC proteolysis [5]. As a control, nonspecific bands detected from polyclonal MgtC antibodies were not affected in all tested conditions (Fig. 2D).

### PhoP Requires FtsH Protease for Its Normal Expression

**Fig. 3:**
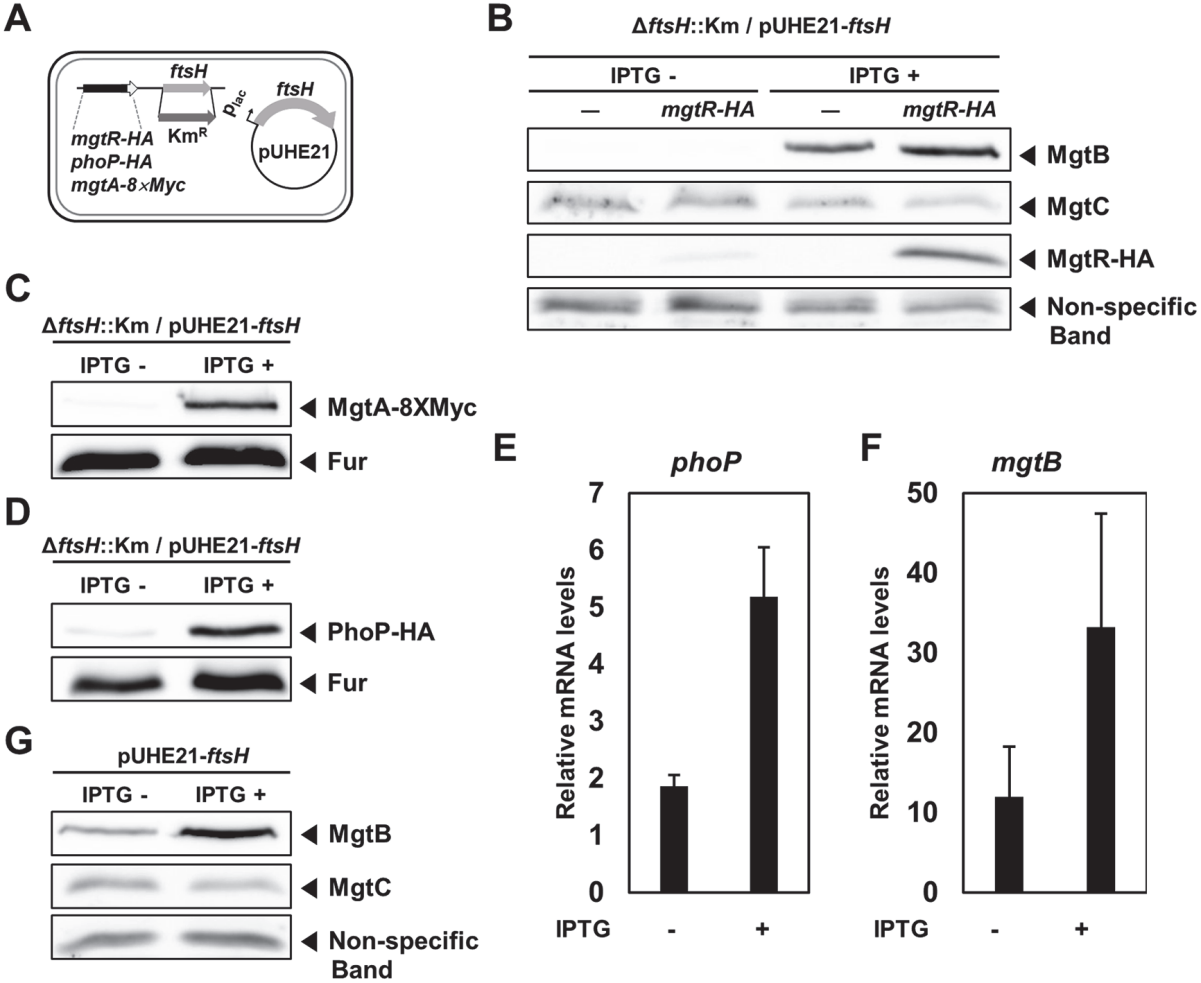
FtsH is required for expression of *phoP* and PhoP-activated genes. (**A**) Schematic cartoon of the *ftsH* conditional knockout mutants with the *mgtR*-*HA* (HK140), *phoP*-*HA* (HK202), or *mgtA*-8×*Myc* (HK164) genes. (**B**) Western blot analysis of crude extracts prepared from the *ftsH* conditional knockout mutants with the wild-type (EN656) or *mgtR*-*HA* (HK140) strains in the absence or presence of IPTG (0.0625 mM). Cells were grown to OD_600_= 0.5 at 37°C in N-minimal medium containing 0.01 mM Mg^2+^. The samples were analyzed by anti-MgtB, anti-MgtC, and anti-HA antibodies to detect MgtB, MgtC, and MgtR-HA proteins. Non-specific bands detected by MgtC polyclonal antibodies were used as loading controls. (**C, D**) Western blot analysis of crude extracts prepared from the *ftsH* conditional knockout mutants with the *mgtA*- 8×*Myc* (C, HK164) or *phoP*-*HA* (D, HK202) strains in the absence and presence of IPTG (0.0625 mM). Cells were grown to OD_600_= 0.5 at 37°C in N-minimal medium containing 0.01 mM Mg^2+^. The samples were analyzed by anti-Myc or anti-HA antibodies. Fur proteins were used as loading controls. (**E, F**) Relative mRNA levels of the coding regions of the *phoP* (**E**) and *mgtB* (**F**) genes produced in the *ftsH* conditional knockout mutant strain (EN656) in the absence and presence of IPTG (0.0625 mM). Cells were grown to OD_600_= 0.5 at 37°C in N-minimal medium containing 0.01 mM Mg^2+^. Expression levels of target genes were normalized to that of 16S ribosomal RNA *rrsH* gene. Relative mRNA levels represent (target RNA/*rrsH* RNA) × 10,000. Shown are the mean and S.D. from two independent experiments. (**G**) Western blot analysis of crude extracts prepared from the wild-type strain harboring pUHE21-*ftsH* plasmid (HK182) in the absence and presence of IPTG (0.0625 mM). Cells were grown to OD_600_= 0.5 at 37°C in N-minimal medium containing 0.01 mM Mg^2+^. The samples were analyzed by anti-MgtB and anti-MgtC antibodies. Non-specific bands detected by MgtC polyclonal antibodies were used as loading controls.

The differential expression patterns of MgtB and MgtC protein levels in the FtsH-depleted strain led to us examine other protein levels controlled by the PhoP/PhoQ two-component system. Firstly, we tested the *mgtR* gene, a third gene in the *mgtCBRU* operon, encoding a regulatory peptide promoting MgtC proteolysis [5]. We created a strain with C-terminally HA-tagged *mgtR* gene in the *ftsH* conditional knockout background (Fig. 3A). Interestingly, MgtR-HA proteins were not detected in the FtsH-depleting condition, similarly to MgtB (Fig. 3B). It implies that the decreases in MgtB and MgtR-HA levels could be due to a change in PhoP levels, controlling transcription of the *mgtCBRU* operon. Still, MgtC protein levels were slightly higher in the FtsH-depleting condition than FtsH-replete condition (Fig. 3B), reflecting that elevated MgtC levels in the FtsH-depleted condition was caused by both decreased MgtR levels and the FtsH-depletion, thus further protecting MgtC from the MgtR-aided FtsH proteolysis.

Secondly, we then tested the *mgtA* gene, another PhoP-regulated gene encoding a Mg^2+^ transporter, located elsewhere in the chromosome [18](Fig. 1B). For that, we created a strain with the C-terminally 8×*Myc*-tagged *mgtA* gene in the *ftsH* conditional knockout background (Fig. 3A). Similarly to what we observed in MgtR-HA levels, MgtA proteins were undetectable when FtsH was depleted (Fig. 3C), indicating that FtsH depletion affects MgtB, MgtR, and MgtA protein levels via an alteration in PhoP levels.

Lastly, to test whether PhoP protein levels are altered by FtsH depletion, we created a strain with the C-terminally HA-tagged *phoP* gene in the same *ftsH* background. In agreement with the previous findings, PhoP-HA protein levels were barely detectable in the FtsH-depleted condition while PhoP-HA proteins were accumulated in the FtsH-replete condition (Fig. 3D). It indicates that FtsH depletion decreases PhoP protein levels, thus affecting transcription of PhoP-controlled genes (Figs. 3E and 3F) and protein levels (Figs. 3B-3D). This phenomenon can be recapitulated even when we expressed the *ftsH* gene from the IPTG-inducible promoter in the wild-type background, not in the *ftsH* conditionally knockout background. MgtB protein levels were lower in the FtsH-uninduced condition than FtsH-induced condition (Fig. 3G). Cumulatively, FtsH protease is necessary for maintaining normal PhoP protein levels and activation of PhoP-regulated genes.

### The PhoP and FtsH Proteins Directly Interact with Each Other

If FtsH is directly involved in PhoP proteolysis, one can expect that PhoP protein levels are higher in the FtsH-depleted condition than the FtsH-replete condition. However, it is unlikely to be the case according to the previous section of data. One possible scenario is that FtsH protein does not degrade PhoP protein but protects PhoP from proteolysis by other proteases such as ClpAP.

**Fig. 4:**
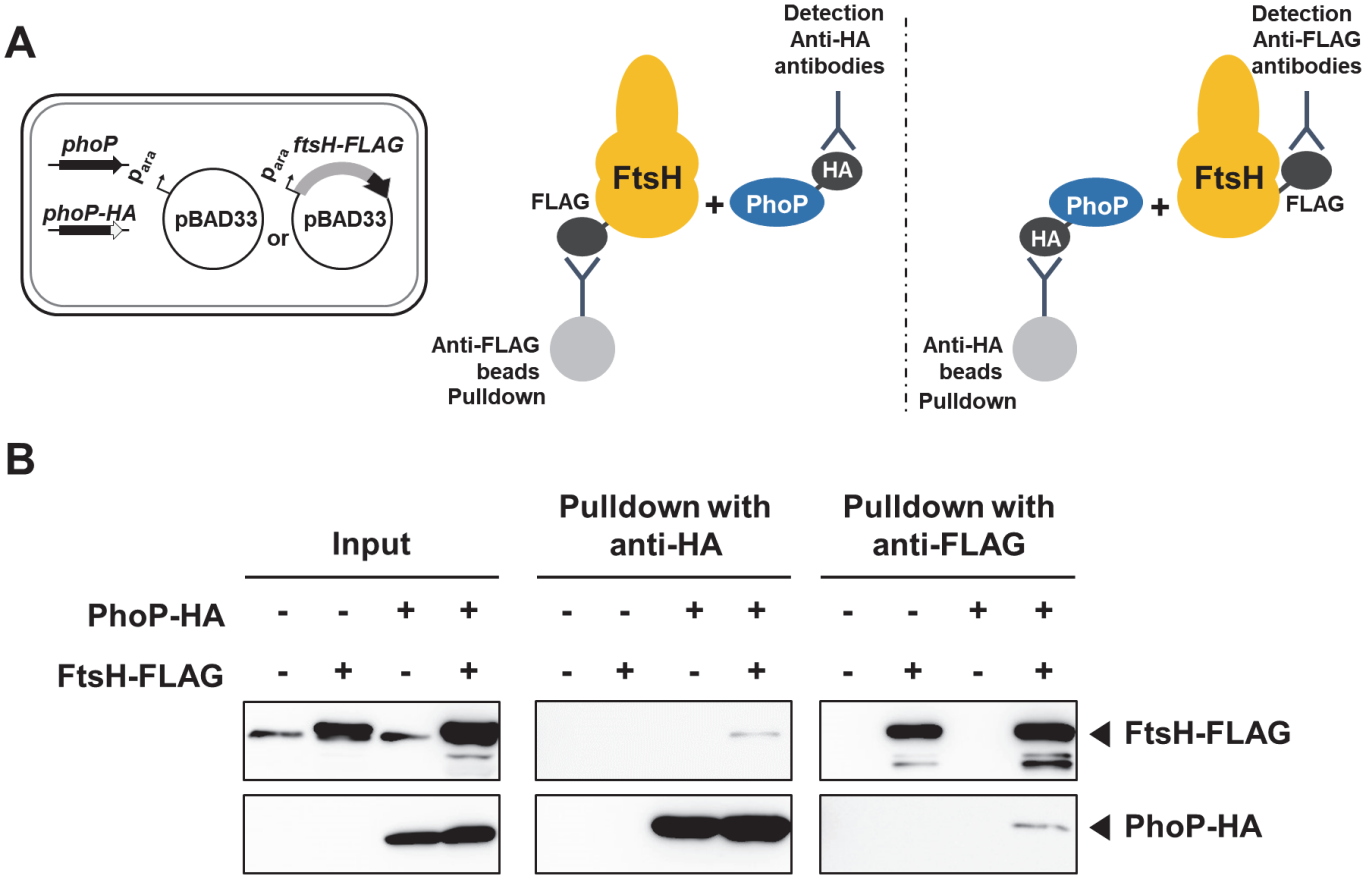
FtsH directly binds to PhoP. (**A**) Schematic diagram of strains used in immunoprecipitation assay between PhoPHA expressed at the chromosomal location and FtsH-*FLAG* expressed from a plasmid. (**B**) A pull-down assay was performed in wild-type strain (no HA tag) with pBAD33 (HK165), wild-type strain (no HA tag) with pBAD33-*ftsH*-*FLAG* (HK166), *phoP*-*HA* strain with pBAD33 (HK167), and *phoP*-*HA* strain with pBAD33-*ftsH*-*FLAG* (HK168). Cells were grown in Nminimal medium containing 0.01 mM Mg^2+^ for 4 h to induce expression of the *phoP*-*HA* gene, and then grown for additional 1 h with 0.5 mM of arabinose for the induction of FtsH-*FLAG*. The lysed cells were incubated with anti-HA or anti-*FLAG* antibody-coated beads overnight. Beads were washed and eluted with 5×SDS sample buffer. The samples were analyzed with anti-HA and anti-*FLAG* antibodies.

To test this possibility that FtsH actually interacts with PhoP, we created a strain with the C-terminally HA-tagged *phoP* gene harboring a plasmid with *ftsH*-*FLAG* gene from an arabinose-inducible promoter. As controls, we used the chromosomal *phoP* gene without HA tag or the empty vector (Fig. 4A). When we pulled down with anti-HA antibodies, the C-terminally HA-tagged PhoP proteins successfully immunoprecipitated FtsH-*FLAG* proteins. Conversely, FtsH-*FLAG* proteins also immunoprecipitated PhoP-HA proteins (Fig. 4B). These data demonstrate that FtsH does not degrade PhoP but controls PhoP protein levels via a direct binding between PhoP and FtsH.

### FtsH Protein Sequestrates PhoP and Protects It from the Proteolysis by ClpAP Protease

**Fig. 5:**
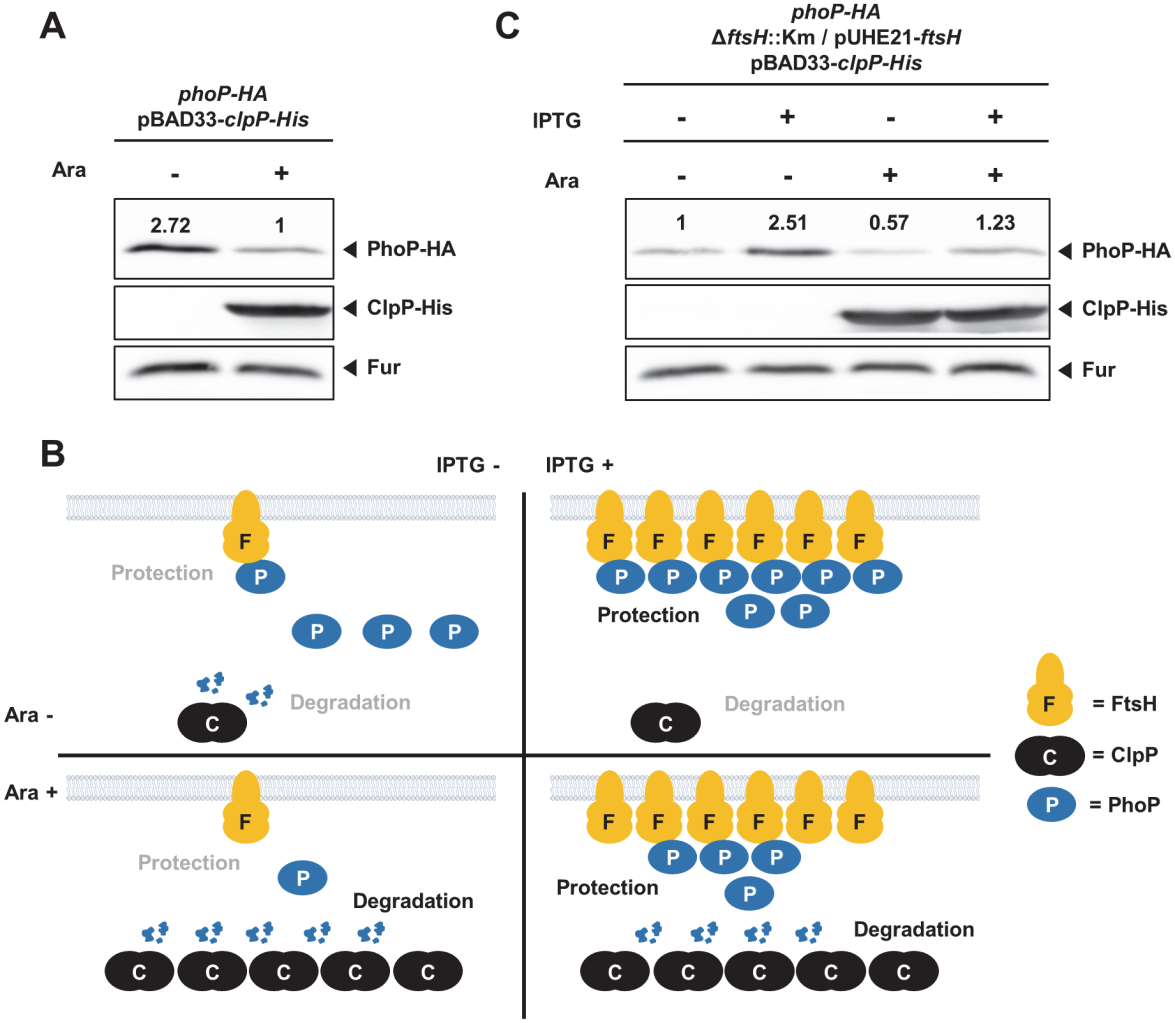
FtsH competes with ClpP protease to protect PhoP from the proteolysis by the ClpP protease. (**A**) Western blot analysis of crude extracts prepared from the *phoP*-*HA* strain harboring pBAD33-*clpP*-*His* plasmid (HK241) in the absence and presence of arabinose (1 mM). Cells were grown to OD_600_ = 0.5 at 37°C in N-minimal medium containing 0.01 mM Mg^2+^. The samples were analyzed with anti-HA and anti-His antibodies. Anti-Fur antibodies were used to detect Fur proteins as a loading control. (**B**) Schematic cartoon of FtsH, ClpP, and PhoP proteins in (**C**). See the text for more details. (**C**) Western blot analysis of crude extracts prepared from the *ftsH* conditional knockout mutant with *phoP*-*HA* gene harboring pBAD33-*clpP*-*His* plasmid (HK242) in the absence and presence of IPTG (0.0625 mM) and arabinose (1 mM). Cells were grown to OD_600_ = 0.5 at 37°C in N-minimal medium containing 0.01 mM Mg^2+^. The samples were analyzed with anti-HA and anti-His antibodies. Anti-Fur antibodies were used to detect Fur proteins as a loading control.

Given that FtsH is required for normal expression of PhoP response regulator and that FtsH physically interacts with PhoP (Fig. 4B), FtsH might protect PhoP proteolysis by sequestrating it from other proteases. ClpP is an immediate suspect based on the previous report that *Salmonella* ClpAP protease degrades the PhoP protein in vitro [14] and ClpP is a catalytic subunit of the ClpAP protease [15]. To examine whether ClpP degrades PhoP proteins, we cloned a C-terminally His-tagged *clpP* gene in an arabinose-inducible plasmid (Fig. 5A). Heterologous expression of the *clpP* gene decreased PhoP protein levels by ~2.7 fold (Fig. 5A), supporting that ClpP protease mediates PhoP proteolysis.

If FtsH binds to PhoP protein and sequestrates PhoP from ClpP protease, excess FtsH protease would compete with ClpP protease for PhoP binding and thus PhoP would be protected from ClpP-mediated proteolysis (Fig. 5B, right top). Conversely, excess ClpP protease would overcome FtsH-mediated sequestration effect on PhoP (Fig. 5B, left bottom). Again, this effect can be reversed by producing more FtsH (Fig. 5B, right bottom). To test this, we created a *phoP*-*HA* strain harboring an arabinose-inducible plasmid with the His-tagged *clpP* gene in the *ftsH* conditional knockout background. We then measured PhoP-HA levels when we added IPTG to produce excess FtsH protease or arabinose to produce excess ClpP protease. Similar to what we observed, FtsH production increased PhoP-HA levels by ~2.5 fold (Fig. 5C). By contrast, excess ClpP production from the arabinose-inducible plasmid decreased PhoP-HA levels by ~2 fold (Fig. 5C). Finally, when FtsH protease was produced in the just-mentioned condition, FtsH seemed to competitively overcome ClpP’s effect and protect PhoP from ClpP-mediated proteolysis, because FtsH production recovered PhoP-HA levels up to ~ 1.2 fold (Fig. 5C). These data demonstrate that FtsH binds to PhoP and competitively protects PhoP proteolysis from ClpP protease.

### Cytoplasmic Domain of FtsH Is Required for PhoP Sequestration

**Fig. 6:**
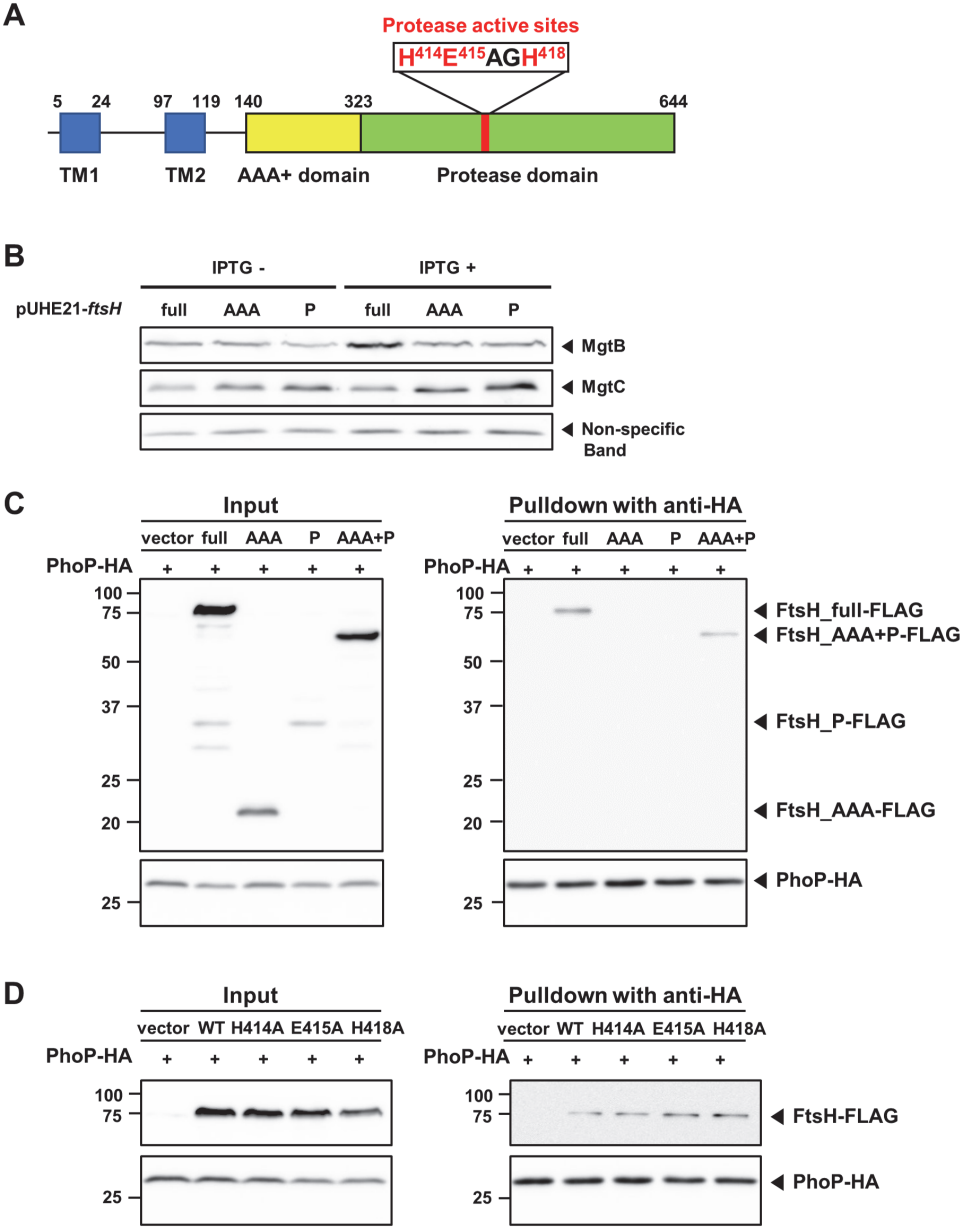
Cytoplasmic domain of FtsH is required for PhoP binding regardless of its catalytic activity. (**A**) Illustration of the domain structure of FtsH. The transmembrane domains (TM1 and TM2; blue), the AAA+ domain (yellow), and the protease domain (green) are indicated. H414, E415, and H418 residues are the proteolytic active sites involved in Zinc binding (red). (**B**) Western blot analysis of crude extracts prepared from bacterial strains harboring the pUHE21 plasmid expressing the full-length FtsH (full), AAA+ domain of FtsH (140-323, AAA), and proteolytic domain of FtsH (324-644, P). Cells were grown with IPTG (62.5 μM) to express the full-length FtsH or each domain of FtsH. Cells were grown to OD_600_ = 0.5 at 37°C in N-minimal medium containing 0.01 mM Mg^2+^. The samples were analyzed with anti-MgtB and anti-MgtC antibodies. Non-specific bands from MgtC blot were used as a loading control. (**C**) A pull-down assay was performed in *phoP*-*HA* strains harboring the pBAD33 plasmid expressing the full-length FtsH (full), AAA+ domain of FtsH (140-323, AAA), proteolytic domain of FtsH (324-644, P), and cytoplasmic domain of FtsH (140-644, AAA+P). Cells were grown in N-minimal medium containing 0.01 mM Mg^2+^ for 4 h to induce expression of the *phoP*-*HA* gene, and then grown for additional 1 h with 0.5 mM of arabinose for the induction of FtsH-*FLAG*. The lysed cells were incubated with anti-HA antibody-coated beads overnight. Beads were washed and eluted with 5× SDS sample buffer. The samples were analyzed with anti-HA and anti-*FLAG* antibodies. (**D**) A pull-down assay was performed in *phoP*-*HA* strains harboring the pBAD33 plasmid expressing the wild-type (HK166) FtsH or FtsH variants with H414A (EN1284), E415A (EN1281), or H418A (EN1282) substitutions. Cells were grown with arabinose (1 mM) to express the wild-type FtsH or FtsH variants. The samples were analyzed with anti-HA and anti-*FLAG* antibodies.

We then wondered which region(s) of FtsH is required for protecting PhoP from proteolysis. FtsH is anchored via two transmembrane helices (TM1 and TM2), and followed by AAA+ domain and protease domain (Fig. 6A)[2]. Because any conditional *ftsH* knockout strain complemented with non-functional or partial *ftsH* gene was expected to be lethal, we tested this in the wild-type *ftsH* background, similar to Fig. 3G. We constructed plasmids harboring either the AAA+ domain or the protease domain under the IPTG-inducible promoter. The *Salmonella* strain expressing the full-length FtsH exhibited an increase in MgtB protein levels, similar to what we observed previously (Fig. 6B). The increase in MgtB protein levels disappeared when we expressed only the AAA+ domain or the protease domain (Fig. 6B), suggesting that neither the AAA+ domain or protease domain is sufficient for FtsH's protecting activity of PhoP protein. Pulldown assay demostrated that PhoP-HA immunoprecipitated full-length or cytoplasmic domain of FtsH but not the AAA+ domain or the protease domain (Fig. 6C), supporting that at least cytoplsmic domain of FtsH is required for PhoP sequestration.

Next, we tested whether FtsH needs to be functional to protect PhoP from proteolysis. FtsH has a conserved HEXXH Zn-binding motif in the protease domain, consisting of the protease active center (Fig. 6A) [2]. Thus, we cloned the full-length *ftsH* gene with substitutions at His414, Glu415, or His418 residues to alanine and expressed in the PhoP-HA background to detect whether those variants bind to PhoP protein. Similarly to the wild-type FtsH-expressing strain, FtsH variants with substitutions were still detected in PhoP-HA-immunoprecipitated eluates (Fig. 6D), indicating that FtsH interacts with PhoP indepedent of its catalytic activity. Therefore, it suggests that cytoplasmic domain of FtsH is required for sequestering PhoP, independent protecting PhoP from proteolysis.

## Discussion

**Fig. 7:**
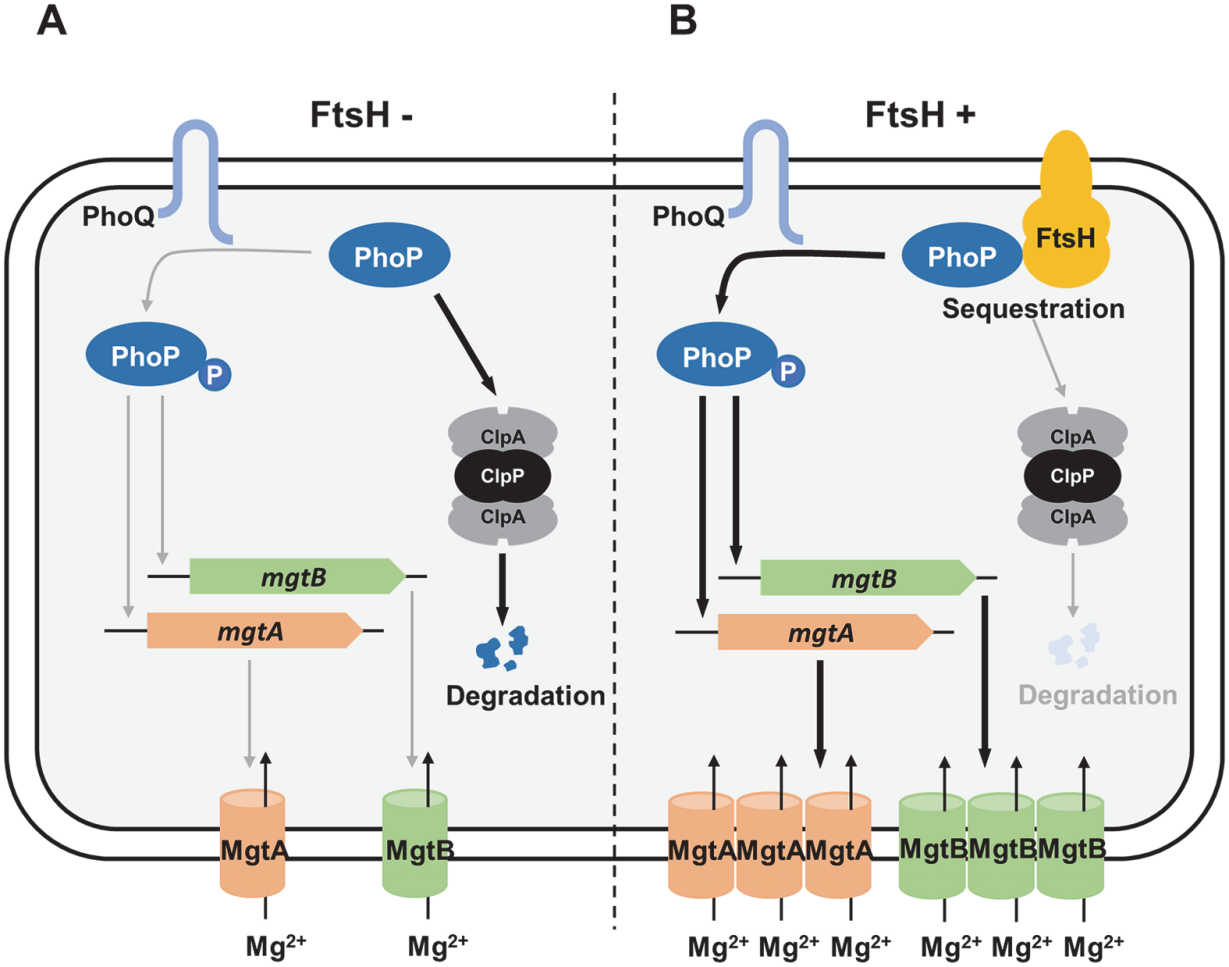
A model for FtsH’s role to protect PhoP from ClpAP protease. (**A**) When FtsH is depleted, PhoP is easily degraded by ClpAP protease thus less amount of PhoP is phosphorylated by PhoQ and levels of PhoP-activated gene products (MgtA and MgtB Mg^2+^ transporters in this graphic) are low. (**B**) When FtsH is replete, FtsH binds to and sequestrates PhoP and protects PhoP from the protease activity of ClpAP. Thus, PhoP protein levels remain high and activate transcription of the PhoP-controlled genes, such as the *mgtA* gene and *mgtCBRU* operon (simplified as *mgtB* in the graphic). Levels of MgtA and MgtB Mg^2+^ transporters maintain high in this condition.

We have demonstrated that *Salmonella* master virulence regulator PhoP protein requires AAA+ protease FtsH for its normal activity as a transcription regulator (Fig. 7). However, unexpectedly from FtsH’s canonical role to breakdown its substrate, FtsH sequestrates PhoP from ClpAP, another AAA+ protease known to break down PhoP protein, and protects PhoP from degradation. This might be beneficial for *Salmonella* to guarantee the amount of PhoP activated by PhoQ during infection. In the absence of FtsH (Fig. 7A), PhoP is degraded by ClpAP protease and subsequently the amount of PhoP protein phosphorylated by PhoQ would be low. Therefore, protein levels of PhoP-activated genes such as the *mgtB* and *mgtR* genes in the *mgtCBRU* operon and the *mgtA* gene were low (Fig. 3). Despite that transcription of the *mgtC* gene is similar to that of the *mgtB* gene, MgtC protein levels increased in the same condition, mainly because of the depletion of MgtR, the small protein promoting degradation of MgtC [5]. If FtsH is intact and available for PhoP (Fig. 7B), FtsH binds to PhoP, preventing the proteolysis of PhoP by ClpAP (Figs. 4B and 5). Under this circumstance, the amount of PhoP activated by PhoQ would be high enough and thus transcript levels of PhoP-activated genes were high subsequently (Figs. 3E and 3F). Because the identified mechanism includes the direct interaction between the membrane-bound FtsH protease and transcription regulator PhoP, molecular details are in question whether FtsH-sequestered PhoP still can be phosphorylated by the cognate PhoQ sensor kinase. It is interesting to note that, similar to MgtC, MgtA Mg^2+^ transporter was suggested to be degraded by the MgtR-mediated FtsH proteolysis because protein levels of MgtA Mg^2+^ transporter decreased by the production of MgtR regulatory peptide [6], which was known to help MgtC proteolysis by the FtsH protease [5]. However, MgtR’s regulatory action toward MgtC that guides FtsH proteolysis seems to be much stronger than that toward MgtA because MgtA levels still decreased in the FtsH-depleted condition even though the MgtR levels became low (Figs. 3B and 3C).

The PhoP/PhoQ two-component system governs expression of genes in response to low extracytoplasmic Mg^2+^ concentration as well as acidic pH and antimicrobial peptides [11]. PhoP protein promotes transcription of genes that aid *Salmonella* to survive in such conditions. Thus, *Salmonella* strain lacking PhoP cannot survive in low Mg^2+^ and replicate within macrophages, which are likely to be low Mg^2+^ [9]. Given that FtsH protease binds to PhoP and protects PhoP from ClpAP proteolysis (Fig. 5), FtsH must be present during *Salmonella* infection to protect PhoP proteolysis by ClpAP, and thus to produce PhoP-regulated genes to survive and replicate within the host. At the same time, among PhoP-regulated genes, the *mgtB* and *mgtC* genes encoding MgtB Mg^2+^ transporter and MgtC virulence factor respectively, were reported previously as the substrates of FtsH protease [4, 5]. Additionally, MgtA Mg^2+^ transporter, another PhoP-regulated gene product, was also suggested to be degraded by the FtsH protease [6]. Based on the facts that FtsH protects PhoP proteolysis from ClpAP protease, but, at the same time, FtsH breaks down several PhoP-controlled downstream gene products including membrane-bound MgtC, MgtB, and MgtA proteins, these opposing roles of FtsH might create a situation, in which PhoP-regulated genes exhibit differential expression patterns in a PhoP-inducing condition.

As FtsH is the AAA+ protease in *Salmonella*, this newly discovered mechanism is the unexpected aspect of FtsH’s activity. Previous studies of FtsH were mainly aimed at searching for the proteolytic substrates of FtsH [2]. We have discovered that FtsH controls cellular physiology not only by degrading its substrate proteins, but also by protecting a certain protein from proteolysis of other proteases. This property is reminiscent of “anti-adaptors” [19], although FtsH is not formally classified into anti-adaptors due to its known function as a protease. One of the formerly reported mechanisms similar to this is anti-adaptor IraP, which enhances the stability of the sigma factor RpoS by sequestering adaptor protein RssB that specifically directs RpoS to ClpXP protease [20, 21]. Similarly, *Salmonella* virulence protein MgtC specifically binds to PhoP and competes with the adaptor protein ClpS, preventing the ClpS to guide PhoP to its protease ClpAP [14]. Here FtsH protease binds to PhoP directly to sequester from ClpAP protease (Fig. 5). Unlike IraP that sequestrates the adaptor protein to prevent the degradation of substrate RpoS, both MgtC and FtsH protect PhoP from its degradation by binding to PhoP and sequestering substrate itself. Another example of adaptor proteins exerting an effect on target protein’s proteolysis includes MgtU. MgtU small regulatory protein binds to MgtB Mg^2+^ transporter and inhibits the proteolysis of MgtB by FtsH protease [4], presumably ensuring the amount of the MgtB protein during infection. However, in the case of MgtU, there is no other known function of MgtU than the regulatory peptide interacting with the MgtB protein. Examples described above are the mechanisms for bacteria to secure the amount/activity of substrate proteins under specific circumstances. Functions of IraP, MgtC, MgtU, and the newly discovered aspect of FtsH have in common that they enhance the stability of substrate proteins via the direct protein-protein interaction with either substrate itself (MgtC, MgtU, and FtsH) or the adaptor protein (IraP). Given that the fine-tuned proteolysis by adaptors and anti-adaptors enables bacteria to resist extracellular stress [22], understanding of these “anti-adaptor-like mechanisms” including FtsH will assist us to gain further insight into the regulation of proteolytic pathway as a bacterial strategy to survive in unfavorable conditions such as inside the host.

## Supplemental Materials

Supplementary data for this paper are available on-line only at http://jmb.or.kr.
