# Supplementary material for: Membrane-Bound Protease FtsH Protects PhoP from the Proteolysis by Cytoplasmic ClpAP Protease in Salmonella Typhimurium
Source: J Microbiol Biotechnol. 2023 Jun 17;33(9):1130–40. doi: 10.4014/jmb.2306.06016 (PMC10580885; doi:10.4014/jmb.2306.06016)
Supplement: Supplementary file 1 [file jmb-33-9-1130-supple.pdf]

J. Microbiol. Biotechnol.
https://doi.org/10.4014/jmb.2306.06016

Supplementary Tables and Figure

Membrane-bound protease FtsH protects PhoP from the proteolysis by cytoplasmic ClpAP protease in *Salmonella* Typhimurium

Hyungkeun Song, Eunna Choi, and Eun-Jin Lee*

Department of Life Sciences, School of Life Sciences and Biotechnology, Korea University, Seoul, Republic of Korea

Table S1. Bacterial strains and plasmids used in this study

Name	Description	Reference
***Salmonella enterica* serovar Typhimurium**		
14028s	wild-type	[1]
MS7953s	*phoP7953*::Tn10	[1]
EN656	Δ*ftsH*::Km^R / pUHE21-*ftsH*	[2]
HK076	Δ*lon*::Km^R	[3]
HK077	Δ*hslUV*::Km^R	[3]
HK078	Δ*clpP*::Km^R	[3]
HK079	Δ*clpXP*::Km^R	[3]
HK080	Δ*clpA*::Km^R	[3]
HK081	Δ*clpP*	[3]
HK082	Δ*clpXP*	[3]
HK083	Δ*lon*	[3]
HK084	Δ*clpA*	[3]
HK087	Δ*hslUV*	[3]
HK094	Δ*ftsH*::Km^R Δ*clpA* / pUHE21-*ftsH*	This study
HK097	Δ*ftsH*::Km^R Δ*hslUV* / pUHE21-*ftsH*	This study
HK118	Δ*clpX*::Km^R	This study
HK122	Δ*clpX*	This study
HK135	*mgtR-HA*	This study
HK140	Δ*ftsH*::Km^R *mgtR-HA* / pUHE21-*ftsH*	This study
HK161	*mgtA-8XMyc*-Km^R	This study
HK162	*mgtA-8XMyc*	This study
HK164	Δ*ftsH*::Km^R *mgtA*-8X*myc* / pUHE21-*ftsH*	This study
HK165	pBAD33	This study
HK166	pBAD33-*ftsH-FLAG*	This study
HK167	*phoP-HA* / pBAD33	This study

HK168	*phoP-HA* / pBAD33-*ftsH-FLAG*	This study
HK182	pUHE21-*ftsH*	This study
HK202	*phoP-HA*, Δ*ftsH*::Km^R / pUHE21-*ftsH*	This study
HK241	*phoP-HA* / pBAD33-*clpP-6XHis*	This study
HK242	Δ*ftsH*::Km^R, *phoP-HA* / pUHE21-*ftsH*, pBAD33-*clpP-6XHis*	This study
EN1573	*phoP-HA*	[4] same as EG13918
EN1284	pBAD33-*ftsH*^{H414A}-FLAG	This study
EN1281	pBAD33-*ftsH*^{E415A}-FLAG	This study
EN1282	pBAD33-*ftsH*^{H418A}-FLAG	This study
HK262	pUHE21-*ftsH_A* (140-323, AAA+ domian)	This study
HK263	pUHE21-*ftsH_P* (324-644, protease domain)	This study
HK265	pUHE21-*ftsH_AP* (140-644, cytoplasmic domain)	This study
HK274	*phoP-HA*, pUHE21-*ftsH_A*	This study
HK275	*phoP-HA*, pUHE21-*ftsH_P*	This study
HK276	*phoP-HA*, pUHE21-*ftsH_AP*	This study
HK251	*phoP-HA*, Δ*clpS*::Km^R	This study
Escherichia coli		
DH5α	*fhuA2 lac(del)U169 phoA glnV44 Φ80' lacZ(del)M15 gyrA96 recA1 relA1 endA1 thi-1 hsdR17.*	[5]
HK240	pBAD33-*clpP-6XHis*	This study
EN1235	pBAD33-*ftsH-FLAG*	This study
EN1278	pBAD33-*ftsH*^{H414A}-FLAG	This study
EN1275	pBAD33-*ftsH*^{E415A}-FLAG	This study
EN1276	pBAD33-*ftsH*^{H418A}-FLAG	This study
HK257	pUHE21-*ftsH_A* (140-323, AAA+ domain)	This study
HK258	pUHE21-*ftsH_P* (324-644, protease domain)	This study
HK273	pUHE21-*ftsH_AP* (140-644, protease domain)	This study
**Plasmids**		
pUHE21-2lacI^q	rep$_{pMBI}$ Ap^R *lacI*^{q}	[6]
pBAD33	pACYC184 *ori* Cm^R	[7]
pKD4	repR$_{6K\gamma}$ Ap^R FRT Km^R FRT	[8]
pKD46	rep$_{pSC101}$^{ts} Ap^R P$_{araBAD}$ γ β exo	[8]
pCP20	rep$_{pSC101}$^{ts} Ap^R Cm^R *cI857* λP$_R$*flp*	[8]
pBOP508	repR$_{R6K}$ Ap^R 8×myc FRT Km^R FRT	[9]

Table S2. Primers used in this study

Name	Sequence (from 5' to 3')	Description
**Knockout, deletion, tagging**		
PHK040	GACGAGTTGCCGCCTTGAT	*clpX* deletion confirm
PHK041	CTCTCTCTTAGTTTAGTGTCCGCC	*clpX* deletion confirm
PHK053	GCATTTGCGTCGTCGTGTGCGGCACAAAGAACAAA GAAGATGTAGGCTGGAGCTGCTTCG	*clpX* deletion Km^R cassette insertion
PHK054	ATCCCCCCTTTTTTGGCTAACTGATTGTATGAATGTT TAACATATGAATATCCTCCTTAG	*clpX* deletion Km^R cassette insertion
PHK057	TGAGGGTCTGCTCTCGCAGA	*mgtR-HA* tagging confirm
PHK058	CTCGCCAACAGCAGCTTATC	*mgtR-HA* tagging confirm
PHK072	TTAGCCTGTTGGTGTTGTGTTTAGCCCTCTGGCAAA TCGTTTTCTATCCATATGATGTTCCAGATTATGCTTA ATGTAGGCTGGAGCTGCTTCG	*mgtR-HA* tagging Km^R cassette insertion
PHK073	TAAAAAATTTTGTCCAACCCTCTTTTTTGCATGGCG TCACCTCGCATATGAATATCCTCCTTAG	*mgtR-HA* tagging Km^R cassette insertion
PHK112	CCGTTAAGCTATTTCCCGTGGC	*mgtA-8XMyc* tagging confirm
PHK113	CCGATCGCGTTAAACTTGTCG	*mgtA-8XMyc* tagging confirm
PHK114	GTTGGTGAAAGGGTTTTACAGCAGACGTTATGGCT GGCAGATCGGATCCAGAATTCGTGAT	*mgtA-8XMyc* tagging Km^R cassette insertion
PHK115	TCGGGGATTAAGCACGCTGGCGAATCCCCGACGAA AGTGTGAGCTCGATCCGTCGACC	*mgtA-8XMyc* tagging Km^R cassette insertion
PHK141	CCATCTGCGGCGTGTTTACC	*clpA* deletion confirm
PHK144	GCTAAGGCCCGGTTTGTACG	*clpA* deletion confirm
PHK155	CACGCCGTTGAATGTGTGG	*lon* deletion confirm
PHK156	GCCTGCCACGCCCTTAC	*lon* deletion confirm
PHK157	CTGAAGATGGCGGGCCATAC	*hslUV* deletion confirm
PHK158	GCGAGGGGTAAGGTTTTGGG	*hslUV* deletion confirm
PHK159	GCGTTCGCGTGAAGTGTAC	*ftsH* deletion confirm
PHK160	GAAAAACCCCGGGGCAAG	*ftsH* deletion confirm
PHK165	CAGGGGGGATCGGGTAAAAATG	*clpS* deletion confirm
PHK166	CCAGATCCACGGAGCATGC	*clpS* deletion confirm

PHK167	TTTTCCTGCCGACAACTGTAACCGATAATGACGACT GACATGTAGGCTGGAGCTGCTTCG	*clpS* deletion Km^{R} cassette insertion
PHK168	GATTGAGCATAGGCACCTCCCCCAATTTTTATACCT GCATCATATGAATATCCTCCTTAG	*clpS* deletion Km^{R} cassette insertion
**Cloning**		
PHK139	GGGGTACCAAAGAGGAGAAATACTAGAATGTCATA CAGCGGAGAACGAG	pBAD33-*clpP-6XHis* Kpn1
PHK140	GCTCTAGAGCTCAGTGGTGATGGTGATGATGATTAC GATGGGTCAAAATTGAGTCAACC	pBAD33-*clpP-6XHis* Xba1
KHU901	GCTCTAGACTTTGTAATGGAACTACATTATGGCGAA AAACCTAATACT	pBAD33-*ftsH-FLAG* Xba1
KHU902	CCCAAGCTTTTACTTGTCATCGTCGTCCTTGTAGTCT TTGTCGCCCAGCTGCT	pBAD33-*ftsH-FLAG* HindIII
KHU893	ACCGCGTACGCCGAAGCGGGC	*ftsH*^{H414A} substitution
KHU894	GCCCGCTTCGGCGTACGCGGT	*ftsH*^{H414A} substitution
KHU895	GCGTACCACGCAGCGGGCCAC	*ftsH*^{E415A} substitution
KHU896	GTGGCCCGCTGCGTGGTACGC	*ftsH*^{E415A} substitution
KHU897	GAAGCGGGCGCCGCGATTATC	*ftsH*^{H418A} substitution
KHU898	GATAATCGCGGCGCCCGCTTC	*ftsH*^{H418A} substitution
PHK169	CGGGATCCATGCGTATGCTGACGGAAGATCAGATC	pUHE21-*ftsH_A* BamH1
PHK170	CCAAGCTTTTAATCCGGCAGGCCTAC	pUHE21-*ftsH_A* HindIII
PHK171	CGGGATCCATGGTGCGCGGTCGTGAG	pUHE21-*ftsH_P* BamH1
PHK172	CCAAGCTTTTATTTGTCGCCCAGCTGC	pUHE21-*ftsH_P* HindIII
**Quantitative real-time PCR**		
6970	CCAGCAGCCGCGGTAAT	*rrsH* qPCR-F
6971	TTTACGCCCAGTAATTCCGATT	*rrsH* qPCR-R
7763	TCAGAAAATGATAAGCAGCATAAAAAA	*mgtB* qPCR-F
7764	CCCTGACGATGGCTGTTCA	*mgtB* qPCR-R
PHK145	GCCTCCCAGGTGATCAACAT	*phoP* qPCR-F
PHK146	TGTATTCGAACGCCGTGAGTT	*phoP* qPCR-R

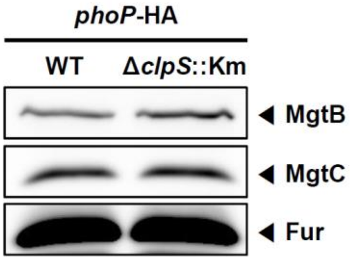

Figure S1. ***clpS*** **deletion does not affect MgtB and MgtC protein levels.**

Western blot analysis of crude extracts prepared from the *phoP-HA* strain (EN1573) and *phoP-HA* Δ*clpS*::Km strain (HK251). Cells were grown to OD_{600}= 0.5 at 37°C in N-minimal medium containing 0.01 mM Mg^{2+}. The samples were analyzed by anti-MgtB, anti-MgtC and anti-Fur antibodies. Anti-Fur antibodies were used to detect Fur proteins as a loading control.

References

1. Fields PI, Swanson RV, Haidaris CG, Heffron F. 1986. Mutants of *Salmonella*-Typhimurium that cannot survive within the macrophage are avirulent. *Proc. Natl. Acad. Sci. USA* **83:** 5189-5193.
2. Choi E, Kwon K, Lee EJ. 2015. A single amino acid of a *Salmonella* virulence protein contributes to pathogenicity by protecting from the FtsH-mediated proteolysis. *FEBS Lett.* **589:** 1346-1351.
3. Kim H, Lee H, Shin D. 2013. The FeoC protein leads to high cellular levels of the Fe(II) transporter FeoB by preventing FtsH protease regulation of FeoB in *Salmonella enterica. J. Bacteriol.* **195:** 3364-3370.
4. Shin D, Groisman EA. 2005. Signal-dependent binding of the response regulators PhoP and PmrA to their target promoters in vivo. *J. Biol. Chem.* **280:** 4089-4094.
5. Taylor RG, Walker DC, McInnes RR. 1993. *E. coli* host strains significantly affect the quality of small scale plasmid DNA preparations used for sequencing. *Nucleic Acids Res.* **21:** 1677-1678.
6. Soncini FC, Vescovi EG, Groisman EA. 1995. Transcriptional autoregulation of the *Salmonella* typhimurium phoPQ operon. *J. Bacteriol.* **177:** 4364-4371.
7. Guzman LM, Belin D, Carson MJ, Beckwith J. 1995. Tight regulation, modulation, and high-level expression by vectors containing the arabinose PBAD promoter. *J. Bacteriol.* **177:** 4121-4130.
8. Datsenko KA, Wanner BL. 2000. One-step inactivation of chromosomal genes in Escherichia coli K-12 using PCR products. *Proc. Natl. Acad. Sci USA* **97:** 6640-6645.
9. Cho BK, Knight EM, Palsson BO. 2006. PCR-based tandem epitope tagging system for *Escherichia coli* genome engineering. *Biotechniques* **40:** 67-72.
